# Primary Ewing’s sarcoma of the intestine: case report and literature review

**DOI:** 10.3389/fonc.2024.1357945

**Published:** 2024-07-30

**Authors:** Baofa Luo, Wei Gao, Ting Li, Xinran Yu, Fei Guo

**Affiliations:** ^1^ Department of Medical Imaging, People’s Hospital of Wenshan Prefecture, Wenshan, Yunnan, China; ^2^ Department of Medical Imaging, The First People’s Hospital of Honghe State, Honghe, Yunnan, China; ^3^ Department of Pathology, People’s Hospital of Wenshan Prefecture, Wenshan, Yunnan, China; ^4^ Department of Oncology, People’s Hospital of Wenshan Prefecture, Wenshan, Yunnan, China

**Keywords:** extraosseous Ewing’s sarcoma, primitive intestine sarcoma, imaging features, clinical manifestations, case report

## Abstract

Ewing sarcoma (ES)/peripheral primitive neuroectodermal tumor is a highly aggressive malignant tumor that typically presents in bone and soft tissue. Primary ES of the intestine is relatively rare, which poses a challenge in distinguishing it from other primary tumors of the small intestine through imaging. This article details a case study of ES originating in the intestine. Computed tomography (CT) imaging suggested a small intestinal stromal tumor, and so the patient underwent resection of the small bowel and omental tumor. Pathology results confirmed the diagnosis of ES of the small intestine. Following surgery, the patient underwent six cycles of chemotherapy, and a follow-up positron emission tomography–CT revealed widespread dissemination of the disease with intraperitoneal metastasis, ultimately resulting in the death of the patient.

## Introduction

Ewing sarcoma (ES)/peripheral primitive neuroectodermal tumor is a highly malignant small round blue cell tumor and the second most common bone tumor in children and adolescents. Extraskeletal ES (EES) has an extremely low incidence of approximately 0.4 cases per million (1). EES predominantly affects the paravertebral region, lower limbs, chest wall, and retroperitoneal area. Cases of primary EES affecting the small intestines are exceptionally rare, with only around 30 reported cases, underscoring the uniqueness of this condition. This article presents a case of ES originating in the small intestine and provides more insights into the clinical features, imaging manifestations, and pathological characteristics of the tumor. By sharing this case, we aim to contribute to a better understanding of this rare disease.

## Case description

**Figure 1:**
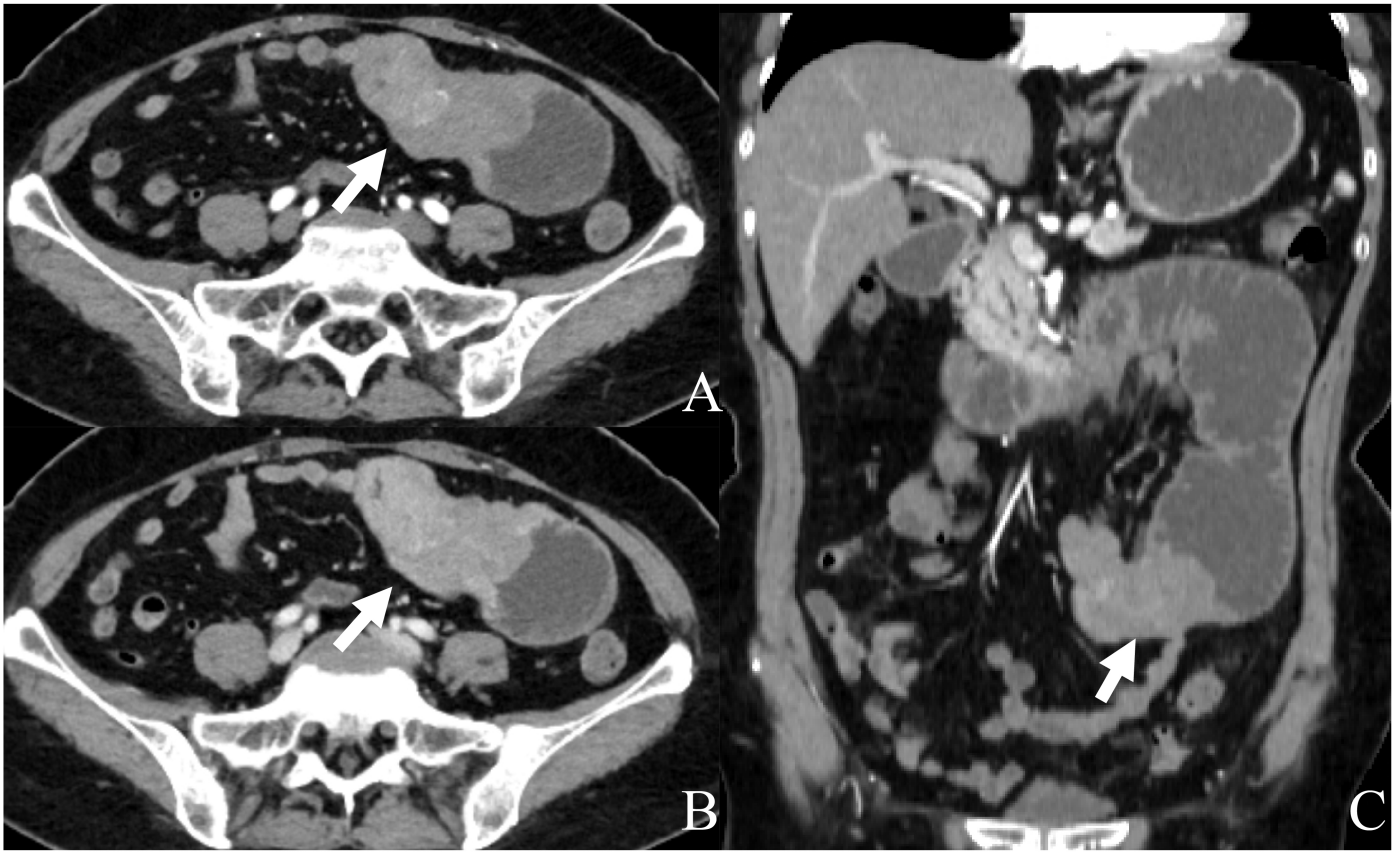
Abdominal CT with contrast: **(A)** axial arterial phase; **(B)** axial venous phase; **(C)** coronal arterial phase. Contrast-enhanced CT displays a well-defined mass of 7.8 cm × 4.2 cm × 5.1 cm (arrow) originating from the small bowel with heterogeneous enhancement, distal intestinal dilatation, and pneumatosis.

A 60-year-old female patient presented with recurrent retrosternal burning sensation, acid reflux, and vomiting with gastric discharge (one to two times/day) persisting for the past 4 months. She received no symptomatic treatment prior to her medical visit. Due to aggravated vomiting (eight times/day) and absence of bowel movements for 5 days, she was admitted to our department. The patient had an unremarkable medical and family history, and physical examination yielded no abnormalities. Laboratory tests for white blood cells, red blood cells, cancer antigen 125 (CA 125), and CA 153 were within the normal ranges. Abdominal computed tomography (CT) revealed intestinal wall thickening, mass-like alterations, and luminal narrowing, with contrast-enhanced CT (arterial phase and venous phase, respectively) showing inhomogeneous enhancement of the mass and absence of swollen lymph nodes in the abdominal cavity (**Figure 1**). The initial impression was small intestinal stromal tumors. Considering the patient’s intestinal obstruction and the challenging nature of pre-surgical biopsy in this anatomical region, an exploratory laparotomy was performed the subsequent day. During surgery, a lesion of 8 cm × 10 cm was identified in the upper jejunum, approximately 40 cm away from the Treitz ligament and occupying 4/5 of the intestinal cavity. Additionally, a mass of 3 cm × 14 cm was found in the ileum about 250 cm away from the Treitz ligament, occupying 2/3 of the intestinal cavity. The mass caused narrowing and stiffness of the intestinal lumen. Tumor deposits were observed in the small intestine mesentery, omentum, and pelvic wall, with no obvious lymph node enlargement. Surgical intervention encompassed tumor resection of the jejunum, ileum, small intestine mesentery, and omentum, with subsequent jejuno-jejunal anastomosis achieved using a linear 75-mm stapler. Post-operative recovery was uneventful.

**Figure 2:**
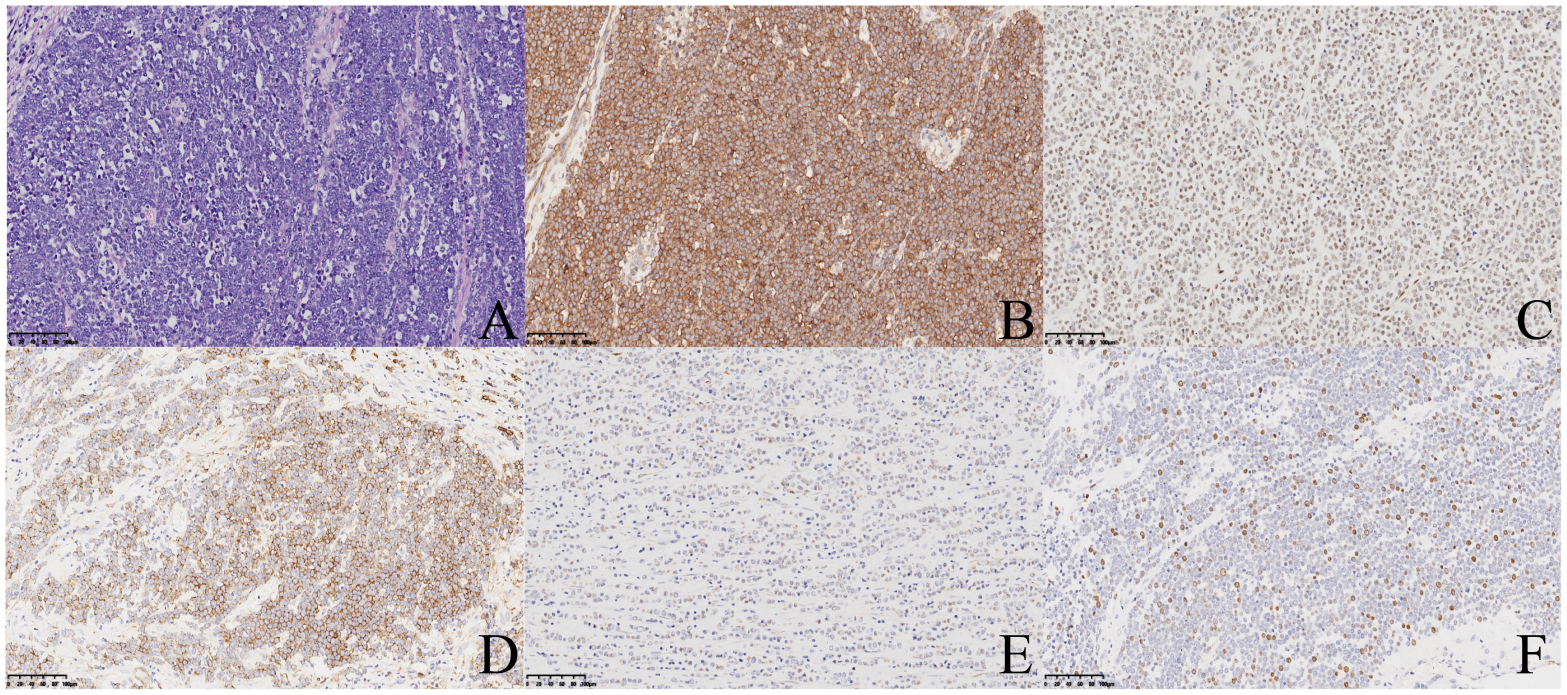
H&E stain **(A)** reveals uniform small round cell; ×20. Immunohistochemistry displays positive staining for CD99 **(B)**, Fli-1 **(C)**, CD138 **(D)**, WT-1 **(E)**, and Ki67 **(F)**; ×20.

**Figure 3:**
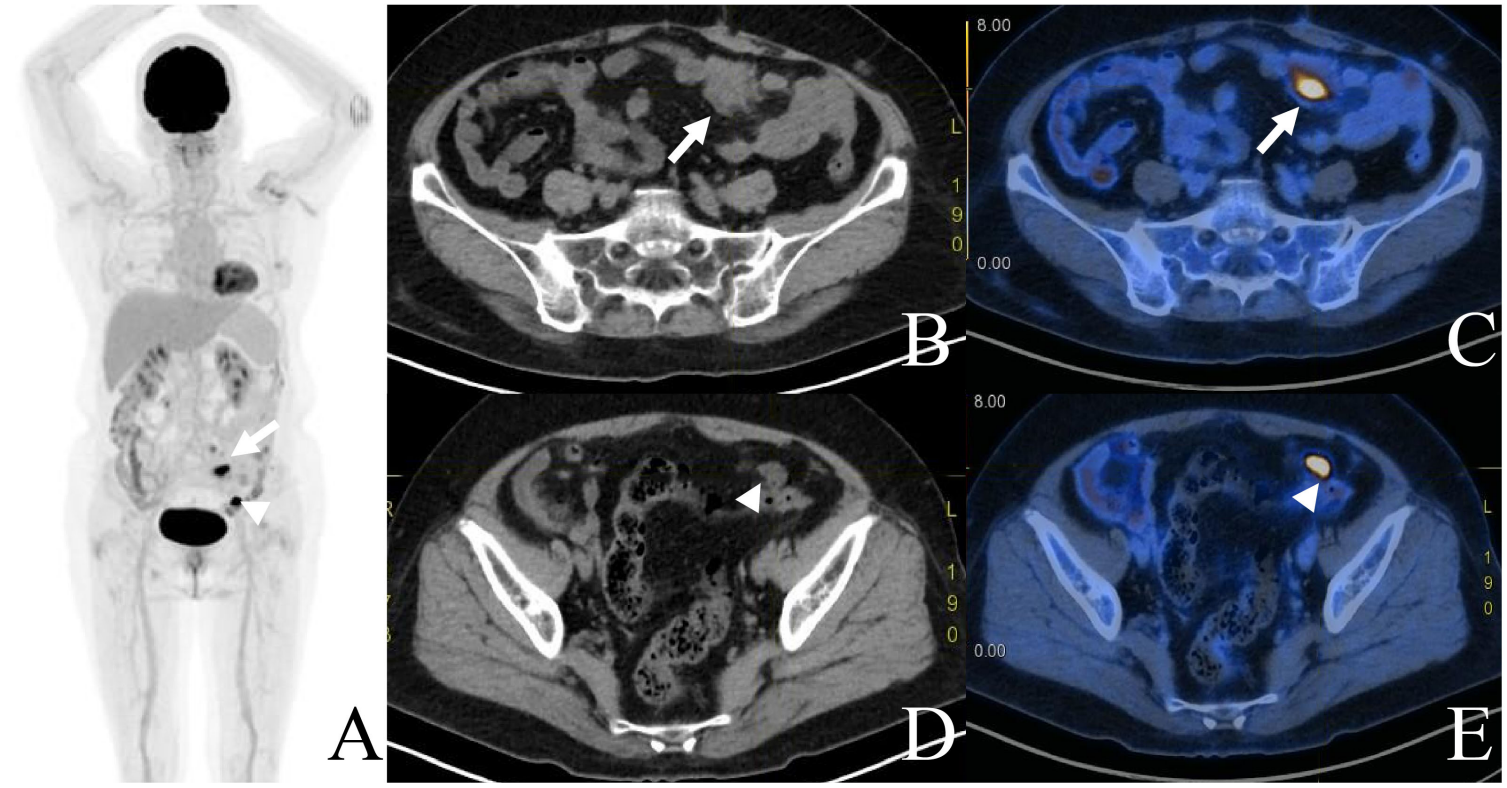
The 18F-FDG PET/CT imaging 11 months after chemotherapy revealed intense uptake in the mesentery (arrows) and peritoneum (arrowheads) around surgical anastomosis, with a SUVmax of 16.3 [**(A)** MIP, **(B, D)** CT, and **(C, E)** fusion].

The gross pathology of the excised jejunal mass consisted of gray-red tumor tissues with serosal involvement. Hematoxylin and eosin (H&E) stain revealed uniformly distributed round and oval tumor cells (**Figure 2A**). Immunohistochemical analysis indicated positive staining for CD99, FLi-1, Ki-67, CD38, and WT-1 and negative for ERG (**Figures 2B–F**). Based on morphology and immunohistochemistry, the patient was diagnosed with ES of the small intestine. The patient underwent eight cycles of adjuvant chemotherapy consisting of VDC (vincristine, doxorubicin, and cyclophosphamide)/IE (ifosfamide and etoposide) given every 3 weeks. Follow-up whole-body 18-fluorodeoxyglucose positron emission tomography (18-FDG PET)/CT imaging performed 5 months post-surgery (two cycles of chemotherapy) revealed no abnormal uptake at the surgical anastomosis site, mesentery, or mesenteric lymph nodes. However, 12 months post-surgery (six cycles of chemotherapy), an MRI revealed a left peritoneal lamellar shadow, corroborated by PET/CT findings indicating metabolic activity in the mesentery and left peritoneal soft tissue nodules, indicative of tumor metastasis (**Figure 3**). Considering that the patient may have extensive peritoneal and intestinal wall adhesions coupled with increased intestinal rigidity, secondary surgery was not considered. Treatment was continued using the VDC/IE regimen. After 2 months of continued treatment, the patient experienced severe myelosuppression and fever, prompting the suspension of chemotherapy. Recombinant human granulocyte colony-stimulating factor was administered to counteract neutropenia, and meropenem was employed for anti-infective management. Upon clinical improvement, the patient was discharged. Three months later, the patient experienced malignant peritoneal effusion, necessitating abdominal puncture and catheterization, followed by carboplatin chemotherapy and oral anlotinib treatment. Nonetheless, the patient developed severe anemia (hemoglobin: 52 g/L), electrolyte imbalance, wheezing, and chest tightness, eventually succumbing to multiple organ dysfunction 19 months post-treatment (**Table 1**).

**Table 1:** Timeline of the case. A 60-year-old female presents with recurrent retrosternal burning sensation, acid regurgitation, and frequent vomiting of gastric secretions (one to two times per day) for 4 months without receiving any treatment. She was admitted to the hospital due to worsening vomiting and absence of bowel movements for 5 days.

Timeline	Diagnostic findings	Interventions
First admission	Laboratory: No obvious abnormalities CT: Neoplastic lesion of the small intestine in the left middle and upper abdomen, mostly considered small intestinal stromal tumor Exploratory laparotomy: Mass of 8 cm × 10 cm in the upper jejunum and mass of 3 cm × 14 cm in the ileum; tumor seeding in the small intestine mesentery, omentum, and pelvic wall	Resection of tumors of the jejunum, ileum, mesentery, and omentum of the small intestine; jejuno-jejunal anastomosis
2 months after surgery	Pathology: Ewing sarcoma of the small intestine	Eight cycles of chemotherapy (VDC/IE)
5 months after surgery (2 cycles of chemotherapy)	PET/CT: No abnormal uptake was seen at the surgical anastomosis site, mesentery, or mesenteric lymph nodes	Continue chemotherapy according to the original plan (VDC/IE)
12 months after surgery (6 cycles of chemotherapy)	MRI: Left peritoneal lamellar shadow PET/CT: Mesentery and left peritoneal soft tissue nodules showing hypermetabolism	Two cycles of chemotherapy (VDC/IE)
14 months after surgery	Severe bone marrow suppression and fever	Terminate chemotherapy; recombinant human granulocyte colony-stimulating factor for leukocyte support; meropenem for anti-infective
17 months after surgery	CT: Mass-like changes in the small bowel in the mid-abdomen, multiple nodular soft tissue density shadows in the peritoneum, mesentery, and abdominal cavity	Carboplatin intraperitoneal perfusion chemotherapy; oral anlotinib
19 months after surgery	Severe anemia; hypoproteinemia; electrolyte disturbances; multiple organ dysfunction	Died

Treatment history and interventions.

## Discussion

ES is a highly malignant tumor typically affecting the long bones and trunk bones. Its manifestation in the small intestine is comparatively rare, with just over 30 reported cases worldwide (2). Given its rarity, specific guidelines for managing ES of the small intestine are lacking, and clinical management typically adheres to guidelines established for ES in general.

We summarize the imaging manifestations, clinical presentation, treatment, and outcomes of primary ES in the small intestine (**Table 2**). Clinical manifestations of intestinal ES mainly include gastrointestinal symptoms. Patients commonly present with abdominal pain, nausea, anemia, and symptoms indicative of intestinal obstruction, whereas, in some severe cases, gastrointestinal perforation may occur due to tumor ulceration (14, 15). Diagnosis of the disease requires imaging methods (CT or MRI), which are capable of clarifying the etiology along with evaluating important information about tumor size, involvement of neighboring structures, and potential metastasis. In this article, we list a total of 12 cases of imaging signs of ES reported in the previous literature (**Table 2**). Small intestinal ES is a highly malignant tumor typically characterized by sizable soft tissue masses in the majority of cases (10/12). Secondary ulceration and/or necrosis may occur as the tumor enlarges (7/12), whereas calcification is a rare finding (1/12). Importantly, inhomogeneous enhancement can be observed after contrast administration. However, these imaging features lack specificity and can lead to confusion with gastrointestinal stromal tumors (GISTs). GISTs typically manifest as submucosal tumors within the gastrointestinal wall, often associated with bleeding, necrosis, or cystic degeneration, and may also lead to intestinal obstruction. Abdominal tumor deposits and hepatic metastasis are common in GISTs, with lymphatic metastasis being infrequent. Therefore, distinguishing between these two conditions based on imaging alone poses a significant challenge, with physicians likely to diagnose a patient with GIST due to the rarity of small intestinal ES.

**Table 2:** Imaging features and clinical manifestations of patients with intestinal Ewing sarcoma.

Author, year	Sex, age	Presenting symptoms	Location	Size (cm)	Radiographic features	Treatment	Outcome
Graham, 2002 (3)	M, 14	Nausea; dizziness	Mesentery of ileum	7 × 7 × 3	Lobulated mass	Surgery + chemotherapy	Sixteen months of DFS
Rodarte, 2012 (4)	M, 32	Lower gastrointestinal bleeding	Ileum	12 × 8	Mass; necrosis	Surgery + chemotherapy	Six months of DFS
Kim, 2013 (5)	M, 35	Abdominal pain	Mesentery of jejunal	11.0 × 6.0	Cystic–solid masses; necrosis	Surgery + chemotherapy	Recurrence of 12 months after diagnosis
Liu, 2016 (6)	M, 15	Abdominal pain; hematemesis; melena	Mesentery of jejunal	17 × 15 × 10	Cystic–solid masses; necrosis	Surgery + chemotherapy	Died 3 months after diagnosis
Li, 2017 (7)	F, 16	Anemia; hematochezia; intestinal obstruction	Ileum	10.0 × 7.3 × 5.3	Mass; necrosis	Surgery	Ten months of DFS
Liao, 2018 (8)	F, 25	Abdominal tenderness; intestinal obstruction	Ileum	9.0 × 6.0	Homogenous mass	Surgery	Lost
Cantu, 2019 (9)	F, 67	Abdominal pain	Jejunal	5.7 × 4.5 × 4.4	Heterogeneous mass; necrosis	Surgery	Lost
Kolosov, 2020 (2)	F, 30	Mild general weakness	Jejunal	4.7 × 6.2 × 6.5	Cystic mass	Surgery	Died 2 months after diagnosis
Shadhu, 2021 (10)	F, 55	No symptoms	Jejunal	3.5 × 3.0 × 2.3	Homogenous mass	Surgery	Three months of DFS
Yang, 2021 (11)	M, 69	Diarrhea; melena	Ileum	6.1 × 3.8 × 4.2	Irregular mass; necrosis	Surgery	Died with metastasis 6 months after diagnosis
Sasaki, 2022 (12)	M, 69	Abdominal pain; vomiting; intestinal obstruction	Jejunal	0.59	Nodular	Surgery	ND
Guo, 2022 (13)	M, 53	Abdominal pain; nausea	Ileum	8.1 × 4.0	Mass; necrosis; calcification	Chemotherapy	Died with metastasis 2 months after diagnosis

F, female; M, male; DFS, disease-free survival; ND, not documented.

The definitive diagnosis of ES relies on pathomorphological features and the immunohistochemical phenotype. ES is characterized by the uniform arrangement of small round bundles or diffuse tumor cells expressing CD99, FLI-1, Ki-67, CD38, and WT-1. While CD99 is highly sensitive in ES (expressed in over 90% of cases) (15), its specificity is relatively low. Approximately 88% of patients with ES exhibit typical chromosomal translocations, particularly the t(11;22)(q24;q12) translocation, which generates the EWS/FLI-1 fusion gene, leading to overexpression of the FLI-1 protein (16, 17). Combining CD99 with FLI-1, a DNA-binding transcription factor involved in the t(11;22) translocation, has been shown to enhance diagnostic efficacy of ES (16). In cases with an unclear diagnosis, confirmation involves detecting EWSR1-FLI1 fusion (85%–95%) and EWSR1-ERG fusion (5%–10%) through fluorescence *in situ* hybridization (FISH) or reverse transcriptase (RT)-PCR analysis (18). FISH is more sensitive for identifying EWSR1 rearrangements than RT-PCR, with a reported sensitivity and specificity of 91% and 100%, respectively, whereas RT-PCR exhibits a sensitivity of 54% and a specificity of 85% (19).

The primary treatment approach for ES involves local surgical excision, with postoperative chemotherapy and radiotherapy aimed at reducing local recurrence. Previous studies have highlighted the preference for surgery over radiotherapy in cases of focal tumors due to concerns regarding the risk of secondary malignancy and radioresistance associated with radiotherapy (20). In addition, advancements in surgical and chemotherapeutic techniques have reduced the reliance on radiotherapy, consistent with data presented in our table (**Table 2**). The first-line systemic chemotherapy regimen usually includes VDC alternating with IE (VDC/IE regimen). Integrated treatment approaches combining multi-agent chemotherapy and surgery have shown significant improvements in prognosis, with a 5-year disease-free survival rate of approximately 65%–70% (21). However, patients with metastasis at diagnosis typically experience a worse prognosis, with an average 5-year survival rate below 30% (22). In cases of metastatic disease, chemotherapy remains an option with limited benefit in extending progression-free survival. In our case, the patient presented with preoperative distant metastases, and, despite undergoing six cycles of systemic chemotherapy, the tumor recurred, ultimately leading to her death.

In summary, ES of the small intestine exhibits high aggressiveness, particularly in elderly patients, leading to a poor prognosis. Imaging plays a crucial role in disease detection and follow-up, typically revealing a large soft tissue mass with narrowing of the affected intestinal lumen. Due to the tumor’s aggressive nature, necrotic and cystic changes may occur within the mass, resulting in uneven enhancement after contrast administration. While imaging aids in disease assessment, the ultimate diagnosis relies on histopathology.

## Data availability statement

The raw data supporting the conclusions of this article will be made available by the authors, without undue reservation.

## Ethics statement

The studies involving human participants were reviewed and approved by People’s Hospital of Wenshan Prefecture (Research Ethics Committee number WYLS2023019). The studies were conducted in accordance with the local legislation and institutional requirements. The participants provided their written informed consent to participate in this study. Written informed consent was obtained from the individual(s) for the publication of any potentially identifiable images or data included in this article.

## Author contributions

BL: Writing – original draft, Writing – review & editing. WG: Data curation, Writing – review & editing. TL: Data curation, Methodology, Resources, Writing – review & editing. XY: Investigation, Project administration, Resources, Writing – review & editing. FG: Conceptualization, Resources, Writing – review & editing.
